# Outbreak of Vaccinia Virus Infection from Occupational Exposure, China, 2017

**DOI:** 10.3201/eid2506.171306

**Published:** 2019-06

**Authors:** Bing Lu, Lun-Biao Cui, Min-Hua Gu, Chao Shi, Chuan-Wu Sun, Kang-Chen Zhao, Jun Bi, Zhong-Ming Tan, Xi-Ling Guo, Xiang Huo, Chang-Jun Bao

**Affiliations:** Wuxi Municipal Center for Disease Control and Prevention, Wuxi, China (B. Lu, C. Shi);; Jiangsu Provincial Center for Disease Control and Prevention, Nanjing, China (L.-B. Cui, K.-C. Zhao, Z.-M. Tan, X.-L. Guo, X. Huo, C.-J. Bao);; Jiangyin Center for Disease Control and Prevention, Wuxi (M.-H. Gu);; Xuzhou Municipal Center for Disease Control and Prevention, Xuzhou, China (C.-W. Sun, J. Bi)

**Keywords:** vaccinia virus, disease outbreaks, orthopoxvirus, vaccination, smallpox, infections, viruses, China, vaccines, occupational exposure

## Abstract

Human infections with vaccinia virus (VACV), mostly from laboratory accidents or contact with infected animals, have occurred since smallpox was eradicated in 1980. No recent cases have been reported in China. We report on an outbreak of VACV from occupational exposure to rabbit skins inoculated with VACV.

Vaccinia virus (VACV; genus *Orthopoxvirus* [OPV]) is used as a lyophilized live virus vaccine against smallpox, variola virus (*1*). VACV, cowpox virus, and monkeypox virus are OPVs of concern because of pathogenicity in humans, possible adverse effects in vulnerable populations, potential spread and introduction in other areas, and public health burden (*2*). After smallpox was declared eradicated in 1980, mandatory routine vaccination was suspended worldwide, including in China (*3*). Immunological cross-reaction herd immunity to OPVs also subsided. Those who might have contact with OPVs often receive VACV vaccination, including those collecting OPV samples, responding to outbreaks, treating patients, or handling OPVs in the laboratory, as well as military personnel, especially in the United States. 

Human infections with OPVs frequently emerge or reemerge, including cowpox virus in Europe (*4*), monkeypox virus in Africa and North America (*5*), buffalopox in India (*6*), and VACV in South America (*7*), especially in Brazil (*8*). Most human cases have occurred from occupational exposure to infected animals or laboratory accidents, such as needle sticks or eye splashes (*9*). No human cases of VACV infection have been reported in China in recent decades.

## The Study

**Figure 1:**
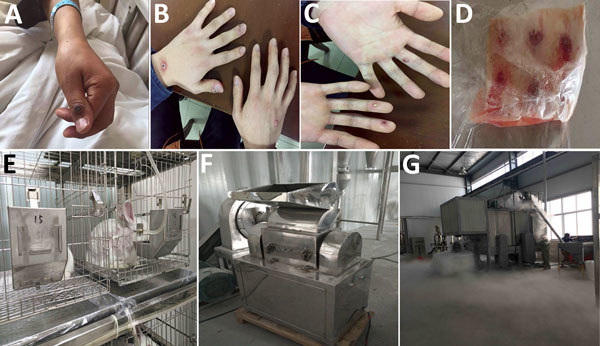
Images from outbreak of vaccinia virus (VACV) from occupational exposure, China, March 2017. A) Lesion on thumb of case-patient 1; B, C) lesions on hands of case-patient 2; D) batch of frozen rabbit skin inoculated with VACV by a biopharmaceutical laboratory; E) live rabbit after VACV inoculation in the biopharmaceutical laboratory; F) machine used to pulverize rabbit skin; G) closed workspace where 5 case-patients pulverized rabbit skin.

On March 9, 2017, case-patient 1, an industrial worker, was admitted to a local hospital with high fever and a pustular eruption on his left thumb that appeared 3 days earlier (Figure 1, panel A). Case-patients 2–5 began having fever and skin lesions almost simultaneously and were seen as outpatients at community clinics during March 7–9. Case-patient 1 became ill with severe pneumonia and was transferred to a tertiary hospital for treatment on March 13.

All 5 case-patients worked in a powder processing company. On March 2, they pulverized a batch of frozen skins (Figure 1, panel D) from rabbits inoculated with VACV (Figure 1, panel E) in a biopharmaceutical laboratory 5 days before. Three biopharmaceutical company employees brought the skins to the powder processing company and used the pulverized skins to study the analgesic function of its extracts. The 5 case-patients pulverized the skins in an enclosed workspace (Figure 1, panel G) and did not wear personal protective equipment at any time during the process, which took ≈1 hour. Case-patients 2, 4, and 5 touched the skins with ungloved hands before and after pulverizing. After the pulverizing process, case-patients 3 and 4 disassembled the pulverizer (Figure 1, panel F), and case-patients 1 and 3 washed it with a water cannon. The 3 biopharmaceutical company employees were in the workspace but wore masks and did not participate in the pulverizing process; none became ill or exhibited symptoms.

On March 14, the municipal Center for Disease Control and Prevention initiated an investigation of the 5 febrile case-patients (Appendix Figure). All 5 case-patients were male, 21–53 years of age (Table). Case-patients 1–4 were otherwise healthy and not taking any long-term medications. Case-patient 5 had a history of hypertension; he had fever but no exanthema. Case-patients 3, 4, and 5 reported having a smallpox vaccination in the 1970s; each had a vaccination scar on his left arm. All case-patients had fevers that began 2–5 days after exposure, with high temperatures of 39.0°C–42.0°C. Case-patients 1, 2, and 4 had painful vesicular-pustular lesions on their hands. Case-patient 2 had >7 pustules with a diameter of ≈0.6 cm on each hand (Figure 1, panels B and C). All 5 case-patients had pulmonary infections seen on computed tomography scans on March 20. 

**Table:** Epidemiologic characteristics in 5 case-patients infected with vaccinia virus from occupational exposure, China, 2017*

Characteristics	Case-patients				
	1 (index)	2	3	4	5
General information					
Age, y	37	21	50	52	53
Sex	M	M	M	M	M
Occupation	Worker	Worker	Worker	Worker	Intermediary
Underlying conditions	N	N	N	N	Y†
Current medications	N	N	N	N	N
Immunosuppressant drugs	N	N	N	N	N
Smallpox vaccination, y	N	N	Y, 1974	Y, 1976	Y, 1977
Vaccination scar	N	N	Y	Y	Y
Date of exposure, March 2017	2	2	2	2	2
Exposure type and duration, min					
Inside enclosed work area	60	60	60	60	30
Contact with rabbit skins, ungloved hand	0	5	0	20	5
Contact with pulverized rabbit skins	0	2–3	0	10	10
Disassembling pulverizer	0	0	30	30	0
Washing pulverizer	5	0	5	0	0
Wearing PPE	N	N	N	N	N
Clinical manifestations					
Date of symptom onset, March 2017	6	6	7	4	6
Date of first doctor visit, March 2017	9	7	8	8	9
Highest temperature, °C; duration, d	42.0; 9	39.2; 7	39.0; 5	42.0; 9	39.2; 8
No. painful vesicular-pustular lesions	1	7	0	3	0
Site of painful vesicular-pustular lesions	Left hand	Both hands	NA	Right hand, torso, upper leg	NA
Other symptoms or laboratory findings	N	N	Headache, muscular pain	Headache, vomiting	Elevated leukocyte count
Complication	Pneumonia	Pulmonary infection	Pulmonary infection	Pulmonary infection	Pulmonary infection
Admitted to hospital	Y	N	N	N	N
Treatment duration, d	16	9	16	10	13
Sequelae of scar formation	N	N	N	N	N
Laboratory results with real-time PCR					
Vaccinia virus (source)	+ (BL, SE, NPS, CS)	+ (BL, SE, NPS, LB)	+ (BL, SE, NPS)	+ (BL, SE, NPS)	–
Cowpox virus	–	–	–	–	–
Monkeypox virus	–	–	–	–	–
*Francisella tularensis*	–	–	–	–	–
*Bacillus anthracis*	–	–	–	–	–

*BL, blood; CS, content of scabs; LB, liquid form blisters; NA, not available; NPS, nasopharyngeal swabs; SE, serum; +, positive; –, negative. †Hypertension and hyperthyroidism.

Case-patients 2–5 were prescribed antipyretic and antiinflammatory medications, which they took for 9–16 days, and were afebrile 5–9 days after illness onset. They were advised to avoid contact with family members and friends until they were afebrile and escharosis exfoliation was complete. Case 1 was the only patient hospitalized. He was kept in isolation in the tertiary hospital until being discharged on March 24. No transmission occurred from the case-patients to other contacts, and none had scar formation. 

Epidemiologic investigation and clinical manifestations raised concerns that cowpox or tularemia were likely etiologies. Nasopharyngeal swab, whole blood, and serum samples were collected from all 5 case-patients on March 14. Clinicians also collected samples of content from scabs from case-patient 1 and liquid from blisters from case-patient 2. 

We extracted viral DNA using the QIAamp MinElute Virus Spin Kit (QIAGEN, https://www.qiagen.com). We conducted real-time PCR to screen for OPV (*10*) and suspected bacteria, including *Francisella tularensis* and *Bacillus anthracis*. Case-patients 1–4 tested positive for VACV. All 5 case-patients were negative for cowpox virus, monkeypox virus, *F. tularensis*, and *B. anthracis*. We used Hep2 cells (SGST accession no. TCHu 21) to isolate the virus in positive samples, which showed characteristic cytopathic effects, including cell rounding, loss of adherence, and cellular debris 48 hours postinfection. 

To establish the relationship between the illness and exposure, we collected residue from the pulverizer blade and outlet and from the surrounding floor. We used real-time PCR to detect VACV-specific nucleic acids in these specimens. We amplified the hemagglutinin gene A56R and sequenced it using an Applied Biosystems 3130 Genetic Analyzer (ThermoFisher Scientific, https://www.thermofisher.com) (*11*). We aligned nucleotide sequences using ClustalW (http://www.clustal.org/clustal2) and constructed a phylogenetic tree with MEGA5 (https://www.megasoftware.net). 

**Figure 2:**
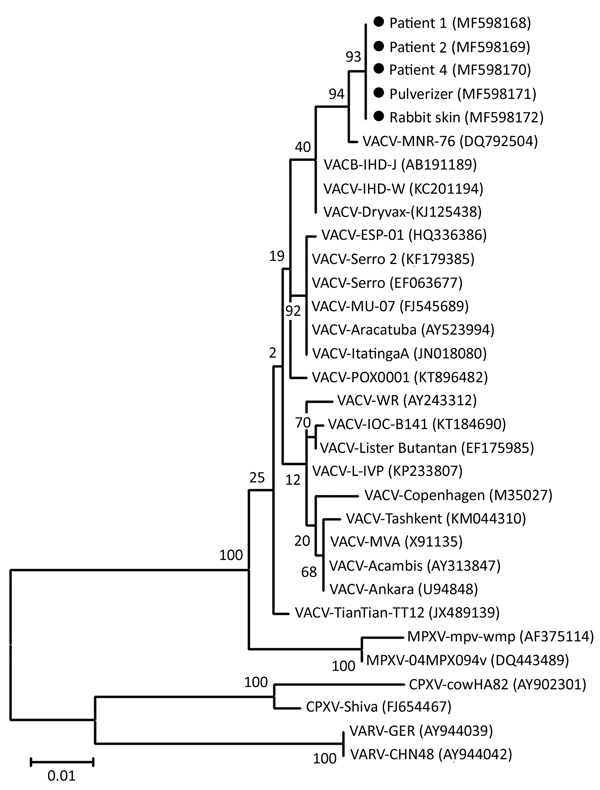
Phylogenetic tree of isolates from outbreak of vaccinia virus from occupational exposure, China, 2017, compared with reference isolates. The tree was constructed by using nucleotide sequences of *Orthopoxvirus* A56R hemagglutinin genes and the maximum-likelihood method with 1,000 bootstrap replicates in MEGA5 (https://www.megasoftware.net). Black dots indicate isolates from this study. Numbers along branches indicate bootstrap values. Scale bar indicates nucleotide substitutions per site. CPXV, cowpox virus; MPXV, monkeypox virus; VACV, vaccinia virus; VARV, variola virus.

We obtained A56R gene sequences from 5 positive specimens, 1 each from case-patients 1, 2, and 4; 1 from the pulverizer; and 1 from the rabbit skin (GenBank accession nos. MF598168–72). All sequences showed 100% identity match with each other and had 99.6% identity match with isolate VACV-MNR-76 (GenBank accession no. DQ792504) (Figure 2). Unfortunately, we could not trace the vaccine strain used to inoculate the rabbits.

## Conclusions

Epidemiologic and molecular data implicated VACV as the etiology of this outbreak, the source of which was rabbit skins inoculated with smallpox vaccine. The phylogenetic tree revealed a close relationship between the isolated strains and the TianTan strain (Figure 2), a highly attenuated VACV strain used in smallpox vaccine in China. The likely transmission route was contact with infected rabbit skins. However, we cannot exclude transmission through aerosolized or suspended infectious particles, considering all 5 case-patients had pulmonary infection and only 3 case-patients had characteristic VACV skin lesions. The transmission mode in this outbreak differs from outbreaks in Brazil and Europe, where human cases were related to contact with infected dairy cattle or to laboratory accidents (*12*–*14*).

Case-patients 3, 4, and 5 received prior smallpox vaccination and had less severe clinical manifestations than the other 2 case-patients, indicating that vaccination might have reduced illness severity but failed to protect them from infection (*13*). Decreased immunity over time and increased virulence of the VACV strain involved might account for the infections. 

VACV is commonly used in research settings and the biopharmaceutical industry. Functional studies on extracts from rabbit skins inoculated with VACV are common in China (*15*). The inadvertent introduction of virulent VACV to local domestic and wild animals could cause a severe outbreak, such as occurred in Brazil (*14*). Besides using standard contact protections, those who handle animals vaccinated with VACV, or their products, should use proper respiratory protection. In addition, we recommend increased supervision of biopharmaceutical uses of VACV and smallpox vaccination for laboratory and other workers at risk for occupational exposure to OPVs, as recommended in the United States (*3*). 

Appendix Additional information on outbreak of vaccinia virus infection from occupational exposure, China, 2017

## References

[1] Essbauer S, Pfeffer M, Meyer H. Zoonotic poxviruses. Vet Microbiol. 2010;140:229–36. 10.1016/j.vetmic.2009.08.026 19828265 PMC9628791

[2] Shchelkunov SN. An increasing danger of zoonotic orthopoxvirus infections. PLoS Pathog. 2013;9:e1003756. 10.1371/journal.ppat.1003756 24339772 PMC3855571

[3] Petersen BW, Harms TJ, Reynolds MG, Harrison LH. Use of vaccinia virus smallpox vaccine in laboratory and health care personnel at risk for occupational exposure to *Orthopoxviruses*–recommendations of the Advisory Committee on Immunization Practices (ACIP), 2015. MMWR Morb Mortal Wkly Rep. 2016;65:257–62. 10.15585/mmwr.mm6510a2 26985679

[4] Maksyutov RA, Gavrilova EV, Meyer H, Shchelkunov SN. Real-time PCR assay for specific detection of cowpox virus. J Virol Methods. 2015;211:8–11. 10.1016/j.jviromet.2014.10.004 25455900

[5] McCollum AM, Damon IK. Human monkeypox. Clin Infect Dis. 2014;58:260–7. 10.1093/cid/cit703 24158414 PMC5895105

[6] Gurav YK, Raut CG, Yadav PD, Tandale BV, Sivaram A, Pore MD, et al. Buffalopox outbreak in humans and animals in Western Maharashtra, India. [PubMed >]. Prev Vet Med. 2011;100:242–7. 10.1016/j.prevetmed.2011.03.008 21511350

[7] de Assis FL, Vinhote WM, Barbosa JD, de Oliveira CH, de Oliveira CM, Campos KF, et al.; Jônatas. Reemergence of vaccinia virus during Zoonotic outbreak, Pará State, Brazil. Emerg Infect Dis. 2013;19:2017–20. 10.3201/eid1912.130589 24274374 PMC3840876

[8] Kroon EG, Mota BE, Abrahão JS, da Fonseca FG, de Souza Trindade G. Zoonotic Brazilian Vaccinia virus: from field to therapy. Antiviral Res. 2011;92:150–63. 10.1016/j.antiviral.2011.08.018 21896287

[9] MacNeil A, Reynolds MG, Damon IK. Risks associated with vaccinia virus in the laboratory. Virology. 2009;385:1–4. 10.1016/j.virol.2008.11.045 19118854

[10] Shchelkunov SN, Shcherbakov DN, Maksyutov RA, Gavrilova EV. Species-specific identification of variola, monkeypox, cowpox, and vaccinia viruses by multiplex real-time PCR assay. J Virol Methods. 2011;175:163–9. 10.1016/j.jviromet.2011.05.002 21635922 PMC9628778

[11] Ropp SL, Jin Q, Knight JC, Massung RF, Esposito JJ. PCR strategy for identification and differentiation of small pox and other orthopoxviruses. J Clin Microbiol. 1995;33:2069–76. 7559950 10.1128/jcm.33.8.2069-2076.1995 PMC228337

[12] Costa GB, Moreno EC, de Souza Trindade G; Studies Group in Bovine Vaccinia. Neutralizing antibodies associated with exposure factors to *Orthopoxvirus* in laboratory workers. Vaccine. 2013;31:4706–9. 10.1016/j.vaccine.2013.08.023 23973323

[13] Hsu CH, Farland J, Winters T, Gunn J, Caron D, Evans J, et al.; Centers for Disease Control and Prevention (CDC). Laboratory-acquired vaccinia virus infection in a recently immunized person—Massachusetts, 2013. MMWR Morb Mortal Wkly Rep. 2015;64:435–8. 25928468 PMC4584810

[14] Abrahão JS, Campos RK, Trindade GS, Guimarães da Fonseca F, Ferreira PC, Kroon EG. Outbreak of severe zoonotic vaccinia virus infection, Southeastern Brazil. Emerg Infect Dis. 2015;21:695–8. 10.3201/eid2104.140351 25811411 PMC4378504

[15] Zhang J, Zhang X, Cui J. Therapeutic evaluation of intradermal injection of inflamed rabbit skin inoculated with vaccinia virus in herpes zoster patients and incidence of post herpetic neuralgia [in Chinese]. Chinese Journal of Experimental and Clinical Virology. 2016;30:566–9.

