# Supplementary material for: Outbreak of Vaccinia Virus Infection from Occupational Exposure, China, 2017
Source: Emerg Infect Dis. 2019 Jun;25(6):1192–5. doi: 10.3201/eid2506.171306 (PMC6537725; doi:10.3201/eid2506.171306)
Supplement: Appendix — Additional information on outbreak of vaccinia virus infection from occupational exposure, China, 2017 [file 17-1306-Techapp-s1.pdf]

Article DOI: https://doi.org/10.3201/eid2506.181306

Outbreak of Vaccinia Virus Infection from Occupational Exposure, China, 2017

Appendix

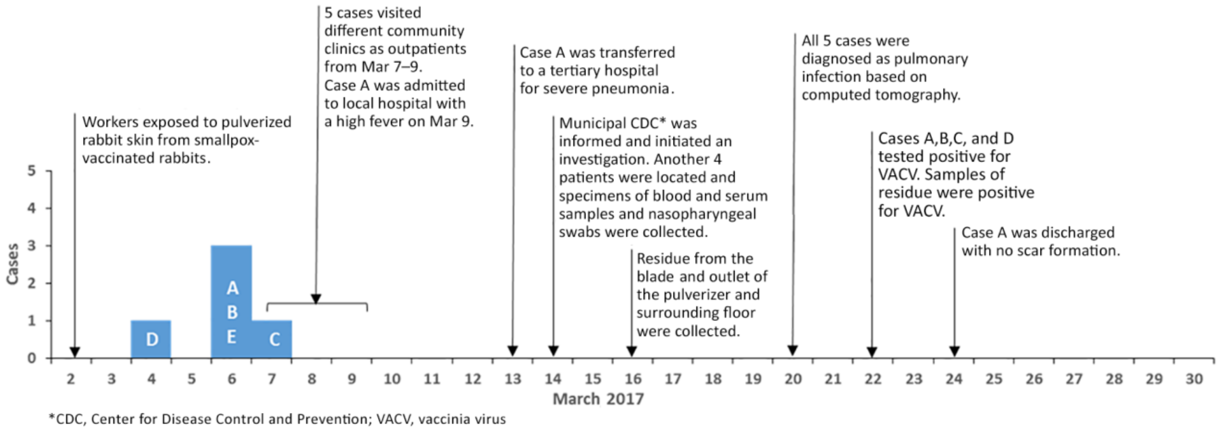

Appendix Figure. Timeline of exposure and outbreak investigation of vaccinia virus infection, China, March 2017. Blue bars indicate onset of symptoms in 5 case-patients, A–E, who pulverized frozen rabbit skins at a powder processing company. The skins were from rabbits inoculated with smallpox vaccine at a biopharmaceutical company 5 days before being brought to the powder processing company.
